# The Institutional Library

**Published:** 1914-12-05

**Authors:** 


### THE INSTITUTIONAL LIBRARY.

### Hahnemann and Modern Medicine.

The Case for Homœopathy. By C. E. Wheeler, M.D., B.S., B.Sc. (The British Homœopathic Association, Chalmers House, 43 Russell Square, W.C. Price 1s.)

Whatever else this small volume may be, it is a model of exposition, good English, and therefore charm of style. We insist on this at the outset because, alas! such virtues do not seem the inevitable results of medical education, and medical literature has suffered much from the lack of them. The volume differs from many controversial works in the fairness with which the case for which it contends is presented, and the final, as the first, plea of Dr. Wheeler is for the test of experiment on the part of his readers of the theory which it propounds. This plea has additional cogency to that of the scientific value of experiment in that homœopathy is so often misstated through ignorance that few students, and certainly not too many older medical men, have been taught to consider carefully what it really is which they are deriding; nor, for instance, to comprehend clearly in what relation the prescribing of castor oil as a remedy for diarrhœa stands to their professed allopathic convictions. Yet such questions as the relation of vaccination and vaccine therapy generally to Hahnemann's theory cannot, as they sometimes are, be brushed aside as not worth considering, except by those to whom the practice of medicine as a professional means of livelihood has overshadowed every other consideration. Moreover, medical men, who fancy that such questions as these are put forward only by those who wish to entrap them into an admission inconvenient to the logic of current medical practice, can take comfort in the similar inquiry as to how far homœopathic hospitals really differ in their theory and practice of treatment from ordinary, not so labelled, institutions. But all this involves genuine scientific interest, much time, and some disturbance of habitual mental processes, and therefore in these busy days, now in particular disorganised through the European war, they are not likely to be particularly popular. Yet we cannot but think, whatever the conclusions arrived at, no medical or institutional reader who has not thrashed out the question before will regret reading this lucid, unprovocative little volume. And, after all, if only we would all realise it, the true recreation for those engaged all day in the art of medical practice is a study of the scientific theories and aspects of medicine itself. Especially in these days of specialism the value of scientific theory needs particular emphasis, lest the immense field of fact and experiment should become, as it were, an overgrown jungle to the mind for the lack of those guiding principles which theories of treatment and disease alone can give.

### Wintering Abroad.

Mediterranean Winter Resorts. By E. Reynolds-Ball, F.R.G.S. Seventh Edition, Revised. With large map of the Mediterranean. Vols. I. and II. together. (Kegan Paul, Trench, Trübner and Co., Ltd. 1914. Price 5s. net.)

This compact handbook contains practical information about a very large number of health resorts on the Mediterranean littoral, including the islands. There is also a section on Egypt and an appendix relating to the West Indies and to India as a winter resort. In addition to concise details about hotels, climate, routes, and sights, there are many short essays by medical men describing the principal resorts from the point of view of the physician. It should be of assistance in helping intending travellers to make a choice, and, although, in view of the very wide field covered by the book, some of the information is necessarily compressed, there is an extraordinary mass of valuable facts available. Most of the information has been derived at first hand, and on the subject of hotels the author has given as full details as one would find anywhere. The map of the Mediterranean to be found in the cover is very full of detail and, though six years old, is quite serviceable; presently, no doubt, a few more drastic alterations will be required. The first appendix contains a veritable *olla podrida* of legal notes, practical hints, notes on living expenses, etiquette, and even medical hints. Some of these items are only misleading in their incompleteness. In fact, the whole book suffers from being crammed unduly.

### A Red Cross Note-book.

Note-book, with Diagrams, for Use at Red Cross Courses of First Aid. (Published for the British Red Cross Society by Henry Frowde and Hodder and Stoughton. 1914. Price 1s.)

A well-printed series of clear diagrams in a note-book of useful size, with plenty of blank space for the student's own notes: such a practical aid in the study of first aid and elementary anatomy is sure to be popular, and we have pleasure in directing attention to its existence. The figures consist in reproductions of diagrams usually shown during the lectures, and thus the student's ofttimes feeble efforts are spared and the time saved in making poor copies can be devoted to the lecturer's explanations and to the filling in of notes and references. The diagrams are purposely left free from any descriptions in order that the student may learn in adding these for himself.

